# Hypoxia potentiates the cytotoxic effect of piperlongumine in pheochromocytoma models

**DOI:** 10.18632/oncotarget.9643

**Published:** 2016-05-26

**Authors:** Petra Bullova, Antony Cougnoux, Luma Abunimer, Juraj Kopacek, Silvia Pastorekova, Karel Pacak

**Affiliations:** ^1^ Section on Medical Neuroendocrinology, Eunice Kennedy Shriver NICHD, NIH, Bethesda, MD, 20892, USA; ^2^ Department of Molecular Medicine, Institute of Virology, Biomedical Research Center, Slovak Academy of Sciences, 84505 Bratislava, Slovakia; ^3^ Section on Molecular Dysmorphology, Eunice Kennedy Shriver NICHD, NIH, Bethesda, MD, 20892, USA

**Keywords:** pheochromocytoma, piperlongumine, hypoxia, reactive-oxygen species, apoptosis

## Abstract

Hypoxia is a common feature of solid tumors that activates a plethora of pathways, resulting in proliferation and resistance of cancer cells to radio- and chemotherapy. Pheochromocytomas/paragangliomas (PHEOs/PGLs) with mutations in the gene coding for the subunit B of succinate dehydrogenase (*SDHB*) are the most aggressive forms of the disease, which is partially due to their pseudohypoxic character, metabolic abnormalities, and elevated reactive oxygen species (ROS) levels. We investigated the effect of piperlongumine (PL), a natural product with cytotoxic properties restricted to cancer cells by significantly increasing intracellular ROS levels, on PHEO cells. Here we report for the first time that PL mediates PHEO cell death by activating both apoptosis and necroptosis *in vitro* and *in vivo*. This effect is magnified in hypoxic conditions, making PL a promising potential candidate for use as a therapeutic option for patients with PHEO/PGL, including those with *SDHB* mutations.

## INTRODUCTION

Hypoxia is a common feature of solid tumors defined by insufficient oxygen levels. Lack of oxygen promotes expression of numerous genes, resulting in several processes including cell proliferation, angiogenesis, migration and metastasis [1]. Expression of hypoxia-related genes is regulated by hypoxia-inducible factors (HIFs) [1]. Pseudohypoxia, on the other hand, is a condition where hypoxia-related pathways are activated despite sufficient levels of oxygen that cannot be properly utilized [2].

Pheochromocytomas (PHEO) are rare catecholamine-producing tumors of neuroendocrine origin located in the adrenal glands. Paragangliomas (PGL) are their extra-adrenal counterparts derived from sympathetic or parasympathetic chromaffin tissue [3]. Thirty five percent of them are linked to germline mutations in several well-described PHEO/PGL susceptibility genes [4]. Based on the recently described concept of HIF signaling alterations in hereditary PHEO/PGL, these tumors have been divided into two clusters. Cluster 1 is characterized by the presence of pseudohypoxia while in cluster 2, HIF signaling is activated through regulation of several pathways, including the PI3K and MAPK pathways [4, 5]. Tumors in cluster 1 include those caused by mutations in genes coding for proteins involved in cell respiration, oxygen level sensors, and Krebs cycle enzymes and their regulators. This cluster also includes the most frequently metastatic tumors caused by mutations in the subunit B of succinate dehydrogenase (*SDHB*) [5].

Apoptosis, autophagy, and necroptosis are the three primary pathways to execute programmed cell death [6]. All three pathways have been shown to be affected by hypoxia through several mechanisms [7–10]. The duration and severity of hypoxia and the phenotype of cancer cells have the most influence on outcome [11, 12]. Moreover, hypoxia is also known to promote epithelial-mesenchymal transition (EMT) and migration, which are both associated with the progression of cancer and metastasis formation [13].

Reactive oxygen species (ROS) are a group of highly reactive molecules under the tight control of intracellular antioxidants. The balance between oxidation and antioxidation is essential for maintaining normal cell functions, as any imbalance leads to a wide range of diseases. Each cell type has a threshold level of intracellular ROS that defines the fate of the cell. While low levels of ROS are beneficial, excessively high ROS levels become cytotoxic and induce cell death [14]. In every cell, low ROS levels are consistently produced in mitochondria during the respiration process, predominantly by the electron transport chain. The redox homeostasis is regulated by antioxidant systems and any ROS level increase leads to an appropriate cell response resulting in elevated antioxidation, converting ROS into non-harmful molecules [15]. ROS are crucial regulators involved in cell adjustments to various stress conditions including hypoxia or starvation; their role is also necessary in stem cell differentiation, immunity and aging [16]. Dysfunctional ROS scavengers, mutations in tumor suppressors, dysfunctional mitochondria or altered metabolic activity lead to ROS accumulation. Depending on the extent of ROS levels increase, one of the two scenarios occurs. In the cells with moderate oxidative stress, elevated ROS levels lead to DNA damage and promote oncogenic transformation [17]. In addition, ROS can act as signaling molecules affecting cellular pathways such as PI3K/AKT, MAPK/ERK1/2 or NF-κB, promoting cancer-related processes including cell proliferation, migration and metastasis, glucose metabolism, differentiation or cell survival [15]. If the antioxidants cannot sufficiently decrease ROS levels, which consequently overcome the ROS threshold the cell is able to maintain, the cell death pathways are activated [14]. Apoptosis and necroptosis are the two main pathways executing cell death induced by ROS [7]. A common feature of cancer cells is a high intracellular level of ROS, suggesting a major role played by ROS in the process of carcinogenesis and cancer progression [18]. The role of hypoxia in increasing intracellular ROS levels is well established [17]. Partially because of pseudohypoxic conditions, high levels of ROS are present in PHEO/PGL, especially in *SDHB*-related tumors, most likely affecting tumorigenesis, as high ROS levels influence HIF signaling [19].

Redox property alterations have become one of the potential strategies of anti-cancer therapies, as cells with elevated ROS levels are more susceptible to oxidative stress-induced cell death [20]. We investigated the effect of piperlongumine (PL), an alkaloid and a proteasome inhibitor naturally synthetized in *Piper longum* L., generally referred to as long pepper, on PHEO cell models (Supplementary Figure S1A). Long pepper has been long used in ayurvedic medicine to treat gastrointestinal and respiratory diseases [21], and in recent years PL has been proved to have numerous effects including antibacterial, antiangiogenic, antimetastatic and antidiabetic. In addition, several studies have shown PL exhibits antitumor activities in various types of cancer cell lines [22]. PL increases ROS levels to a high extent and exceeds the threshold compatible with cell survival. PL has been observed to promote cell death by activating several mechanisms including apoptosis, autophagy, and necrosis affecting PI3K/AKT/mTOR, p38/JNK, MEK/ERK, and NFκB pathways [21, 23–25]. In addition, PL-induced cytotoxity is selective and the compound kills predominantly cancer cells without affecting normal cells, making PL a good candidate for cancer treatment [26]. To our knowledge, increasing ROS levels have never been evaluated as a possible treatment option in PHEO/PGL. We show that PL induces ROS-dependent apoptosis and inhibits cell migration *in vitro* and metastasis formation in a PHEO allograft mouse model. Moreover, this is the first report showing the necroptosis-inducing effect of PL and necroptosis in PHEO in general. Furthermore, this report shows for the first time that the effect of PL is potentiated by (pseudo)hypoxia, making this compound a promising agent for cancer therapy in patients with PHEO/PGL, including *SDHB*-related tumors.

## RESULTS

### Piperlongumine exhibits a ROS-dependent pro-apoptotic and pro-necroptotic effect on mouse pheochromocytoma cell models

**Figure 1:**
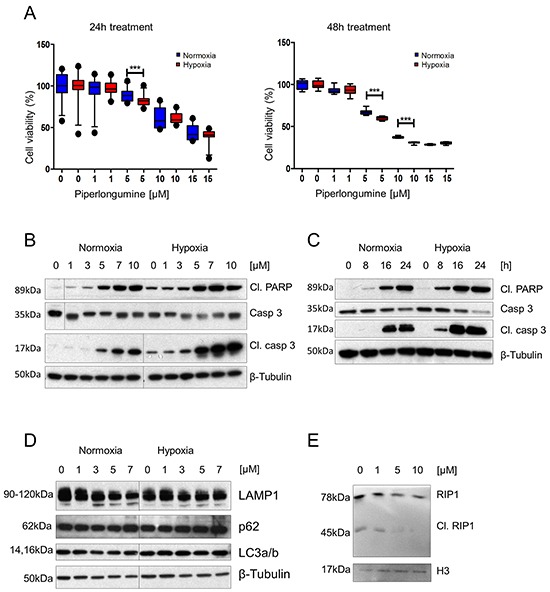
PL exhibits a cytotoxic effect on PHEO cell models via apoptotic and necroptotic pathways, and this effect is enhanced by hypoxia **A.** MPC cells were treated with 0, 1, 5, 10, and 15μM PL at 21% and 1% O_2_ for 24 and 48 hours. Cell viability was assessed by MTT assay. The box and whiskers graphs represent data from three independent experiments. **B.** MPC cells were treated with the indicated concentrations of PL at 21% and 1% O_2_ for 24 hours. Total cell lysates were subjected to Western blot with antibodies against cleaved PARP, cleaved caspase 3, and caspase 3. β-tubulin was used as a loading control. A representative image (n=3) is shown. **C.** Time course analysis of cleaved PARP, cleaved caspase 3, and caspase 3 in cell lysates from MPC cells treated with 10μM PL at 21% and 1% O_2_. β-tubulin was used as a loading control. A representative image (n=3) is shown. **D.** MPC cells were treated with indicated concentrations of PL at 21% and 1% O_2_ for 24 hours. Total cell lysates were analyzed by Western blot for p62, LAMP1 and LC3a/b. β-tubulin was used as a loading control. The representative image (n=3) is shown. **E.** MPC cells were treated with indicated concentrations of PL for 24 hours. Total cell lysates were analyzed by Western blot for cleaved RIP1. Histone 3 was used as a loading control. The representative image (n=3) is shown. ***P<0.001, Mann Whitney, U-test. Cl: cleaved. PL: piperlongumine.

We first performed a viability assay to assess the effect of PL on MPC cells. PL exhibited its cytotoxic effect in a dose- and time-dependent fashion, with 38.5±2.58% and 63±0.31% of dead cells at 24 and 48h (IC_50_=6.04μM), respectively, at 10μM PL (Figure 1A; Supplementary Figure S1B). MTT cells showed a similar outcome after 48h treatment (IC_50_=4.8μM) (Supplementary Figure S1C).

Next, we investigated which pathways were involved in PHEO/PGL cell cytotoxicity. We observed significantly increased levels of the apoptosis markers cleaved caspase 3 and cleaved PARP upon PL treatment. The measured increase in apoptosis markers was dose- and time-dependent (Figure 1B and 1C). Levels of the autophagy-associated proteins p62, LAMP1, and LC3a/b did not exhibit any change in the presence of PL at any concentration (Figure 1D). In addition, we evaluated necroptosis-associated proteins – receptor-interacting protein kinase 1 and 3 (RIP1 and RIP3). While RIP1 is involved in activation of apoptotic, NF-κB and necroptosis pathways, RIP3 is associated with necrosis. Both kinases are essential parts of the necrosome complex responsible for execution of necroptosis [27, 28]. In a cell undergoing apoptosis, RIP1 and RIP3 are cleaved, and thus inactivated, by caspase 8. However, absence of the cleaved RIP1 and RIP3, and their participation in the necrosome is a hallmark of necroptosis [28]. We found decreased levels of cleaved RIP1 at 5 and 10μM PL in MPC cells, suggesting that necroptosis was taking place in these cells as well (Figure 1E).

**Figure 2:**
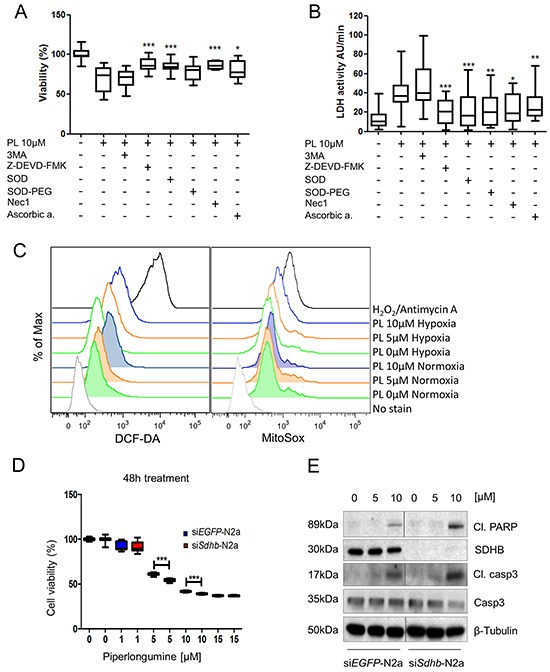
The cytotoxic effect of PL is induced by ROS increase and it is magnified in (pseudo)hypoxia **A.** MPC cells were pre-treated with 3MA, for 4 hours and Z-DEVD-FMK, SOD, SOD-PEG, Nec1 and ascorbic acid for 1 hour before treatment with 10μM PL for 48 hours. Cell viability was assessed by MTT assay. The box and whiskers graphs represent data from three independent experiments. **B.** Absorbance was measured every minute over a 10-minute period to investigate activity of LDH and assess leakage of LDH. The box and whiskers graphs represent data from three independent experiments. **C.** MPC cells were treated with 0, 5, and 10μM PL at 21% and 1% O_2_ for 3 hours. ROS production levels were analyzed by flow cytometry. Treatment with hydrogen peroxide was used as a positive control. A representative image (n=3) is shown. **D.** N2a cells were transfected with 20nM siRNA targeting *Sdhb* or *EGFP* (control). They were treated with 0, 1, 5, 10, and 15μM PL for 48 hours. Cell viability was assessed by MTT assay. The box and whiskers graph represents data from three independent experiments **E.** N2a-*siSdhb* and N2a-*siEGFP* cells were treated with indicated concentrations of PL for 24 hours. Total cell lysates were subjected to Western blot with antibodies against cleaved PARP, cleaved caspase 3, SDHB and caspase 3. β-tubulin was used as a loading control. The representative image (n=3) is shown. *P<0.05; **P<0.01; ***P<0.001, Mann Whitney, U-test. Cl: cleaved. PL: piperlongumine; DCF-DA: 2′,7′-dichlorofluorescin diacetate.

To further confirm the results, MPC cells were treated with the cleaved caspase 3 inhibitor Z-DEVD-FMK; 3-methyladenine (3MA), a well-known autophagy inhibitor; and Necrostatin 1 (Nec1), a necroptosis inhibitor [29]. The results showed a significant protective effect of Z-DEVD-FMK and Nec1 on cells treated with PL by 14.5±2.02% SEM and 14.2±1.44% SEM, respectively, while 3MA did not interfere with the cytotoxic effect of PL on MPC cells, confirming that death of these cells is mediated through both apoptosis and necroptosis (Figure 2A, 2B). We also determined the ubiquitin profile and ROS levels in MPC cells, as PL has been shown to inhibit the 26S proteasome and increase ROS levels in various cancer cells [30]. PL had no effect on the ubiquitin profile in MPC cells (Supplementary Figure S1D). Levels of ROS were elevated with increasing concentrations of PL after 3h of treatment (Figure 2C; Supplementary Figure S1E), suggesting that apoptosis/necroptosis is induced by high ROS levels in PHEO cells. To confirm that ROS are responsible for activation of cell death, we treated MPC cells with PL in the presence of ROS scavengers. Cells treated with the recombinant superoxide dismutase (SOD) in addition to PL showed significantly higher cell viability (85±1.15%) when compared to the cells treated with PL alone (72±2.42%). Superoxide dismutase-PEG (SOD-PEG) did not exhibit a significant protective effect on the cells treated with PL when analyzed by MTT assay, but it significantly decreased lactate dehydrogenase (LDH) leakage (Figure 2A, 2B). A similar protective effect was achieved by pre-treatment with ascorbic acid. The data suggests that PL induces apoptosis and necroptosis in MPC cells by increasing intracellular ROS.

### Hypoxia increases the cytotoxic effect of piperlongumine

**Figure 3:**
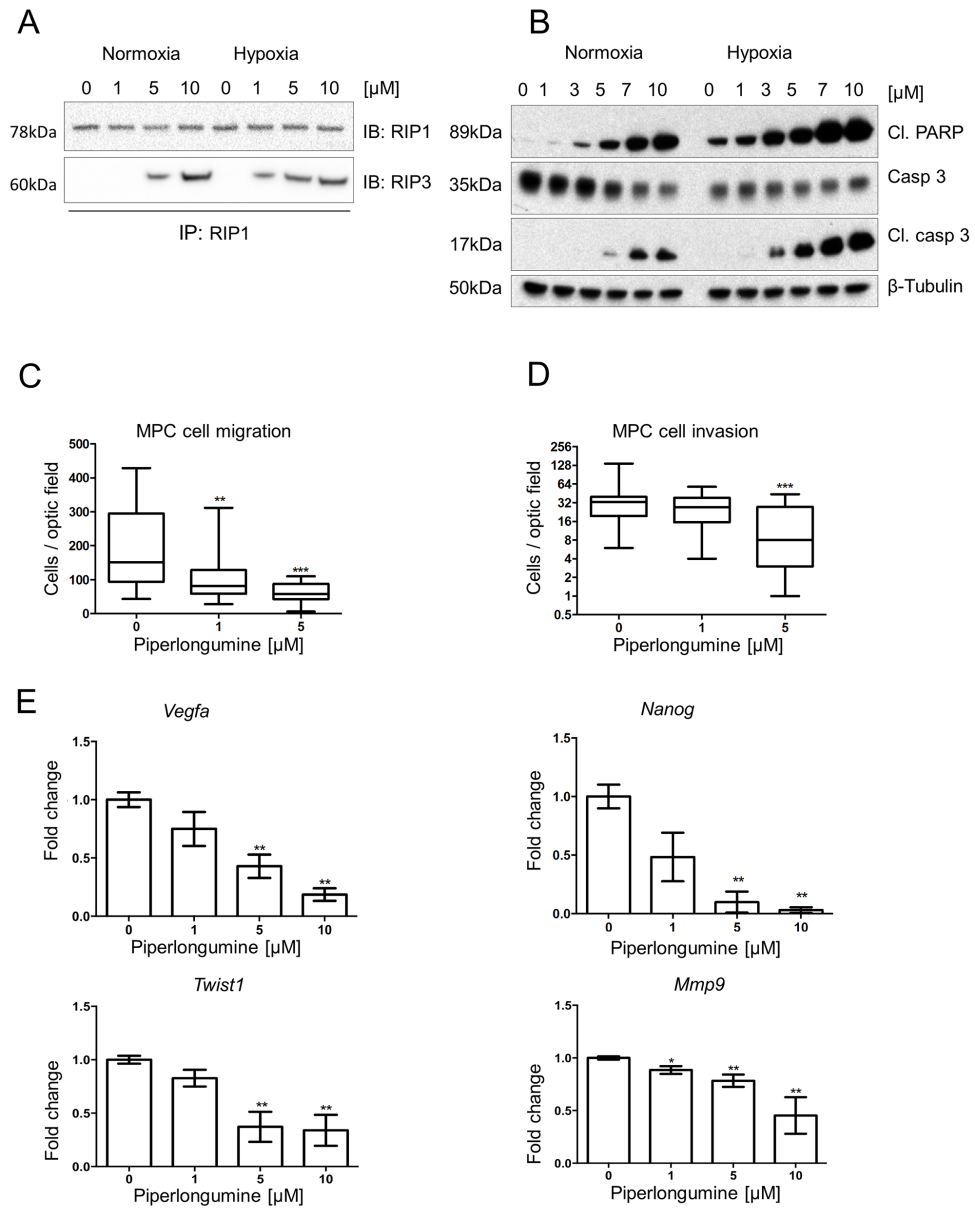
Piperlongumine activates apoptosis and necroptosis, and inhibits cell migration and invasion **A.** RIP1-IP was performed on cell lysates from MPC cells treated with the indicated concentrations of PL at 21% and 1% O_2_ for 24 hours and probed for RIP1 (top lane) and RIP3 (bottom lane). A representative image (n=3) is shown. **B.** MTT cells were treated with indicated concentrations of PL at 21% and 1% O_2_ for 24 hours. Total cell lysates were analyzed by Western blot for cleaved PARP, cleaved caspase 3 and caspase 3. β-tubulin was used as a loading control. The representative image (n=3) is shown. **C.** 1.5 × 10^5^ MPC cells were plated in the upper part of transwell chambers and allowed to migrate for 24 hours in the presence of 0, 1, and 5μM PL. The box and whiskers graph represents data from three independent experiments. **D.** 1.5 × 10^5^ MPC cells were plated in the upper part of matrigel-coated transwell chambers and allowed to migrate for 24 hours in the presence of 0, 1, and 5μM PL. The box and whiskers graph represents data from three independent experiments. **E.** MPC cells were treated with indicated concentrations of PL for 24 hours. mRNA expression levels of *Twist1, Vegfa, Mmp9,* and *Nanog* were assessed by quantitative real-time PCR. The target gene transcript levels were normalized to *Actb*. A graph represents data from three independent experiments as mean +/− SEM. *P<0.05; **P<0.01, ***P<0.001, Mann Whitney, U-test. *Mmp9*: matrix metallopeptidase 9; Cl: cleaved; PL: piperlongumine; IB: immunoblot; IP: immunoprecipitation.

Cells in hypoxic areas of solid tumors are frequently associated with resistance to chemo- and radiotherapy [1]. Thus, we tested the effect of PL on MPC cells in hypoxia. Viability assays showed that the cytotoxic effect of PL on the cells was increased in hypoxia when compared to normoxic cells (Figure 1A). MPC cells exhibited less viability in hypoxia at a concentration of 5μM PL after 24h, and 5 and 10μM after 48h. Currently, there is no *SDHB*-mutated PHEO/PGL cell line available; therefore, we used N2a cells, which are derived from mouse neuroblastoma. Neuroblastoma-derived cells are frequently used in studies of PHEO/PGL as neuroblastoma is a tumor of neural crest origin that shares numerous similarities with PHEO/PGL [31, 32]. A viability assay was performed on N2a cells with silenced *Sdhb* (si*Sdhb*-N2a) to model tumors lacking functional SDHB, similar to *SDHB*-related PHEO/PGL (Supplementary Figure S1F, S1G). The percentage of viable cells at 5μM PL after 48h was significantly lower in si*Sdhb*-N2a (54±0.36%) than in control si*EGFP*-N2a cells (61±0.3%) (Figure 2D, Supplementary Figure S1H). We also investigated ROS levels in hypoxia and exposed MPC cells with or without PL to either normoxia or hypoxia for 3h. The results confirm that hypoxia itself increases intracellular ROS levels and the combination of low oxygen levels and PL further increased levels of ROS in MPC cells (Figure 2C; Supplementary Figure S1E). To evaluate whether hypoxia also increases activation of apoptosis, we analyzed MPC cells treated with PL in normoxic and hypoxic conditions (Supplementary Figure S2A). As hypothesized, levels of cleaved caspase 3 and cleaved PARP were higher in hypoxic cells, showing an increase in apoptosis induction beginning at 3μM PL (Figure 1B). Similar results were observed in N2a cells where levels of apoptosis markers were higher in *siSdhb*-N2a than in *siEGFP*-N2a (Figure 2E). We also evaluated the accumulation of cleaved caspase 3 and cleaved PARP in MPC cells over time. While in normoxia we detected an increase of cleaved caspase 3 and PARP after 16h, in hypoxia similar activation was observed after only 8h of treatment (Figure 1C). In addition, we observed a necrosome assembly in cells treated with PL for 24h at 5μM PL in normoxia, while in hypoxia the effect was evident at only 1μM (Figure 3A). Again, there was no difference in the autophagy markers at any concentration (Figure 1D). MTT cells showed the same pattern when treated with PL in normoxia and hypoxia (Figure 3B). To suggest which signaling pathways are involved in the PL-induced cell death, we performed a phospho-kinase array on MPC cells treated with 10μM PL in normoxia and hypoxia for 24h. We observed an activation of ERK-1/2, p38α, and JNK1/2/3, all known transducers of ROS-associated signaling pathways [33] (Supplementary Figure S2B). Interestingly, activation of β-catenin and GSK-3α/β, proteins involved in cell proliferation and migration [34], was inhibited in hypoxic cells upon PL treatment. These results suggest that hypoxia enhances the cytotoxic effect of PL on PHEO cells.

### Piperlongumine inhibits cell migration and invasion

We analyzed PL influence on PHEO cell migration and invasion, early steps of processes leading to metastases formation. We observed a significant inhibition of migration in cells treated with 1 and 5μM PL compared to controls (Figure 3C). We observed a similar effect of PL on invasion, where significant decrease in the number of invading MPC cells was observed at 5μM PL (Figure 3D). Next, we investigated whether the reduced migration/invasion was due to targeting the EMT and angiogenic properties of these cells. We observed a significant decrease in expression of *Mmp9, Vegfa1, Twist1,* and *Nanog* at 5μM and 10μM PL after 24h treatment (Figure 3E). These results suggest that PL inhibits migration and invasion of PHEO cells, and thereby might reduce their metastatic propensity *in vivo.*

### Piperlongumine inhibits tumor growth in vivo and suppresses angiogenesis, metastases and EMT

**Figure 4:**
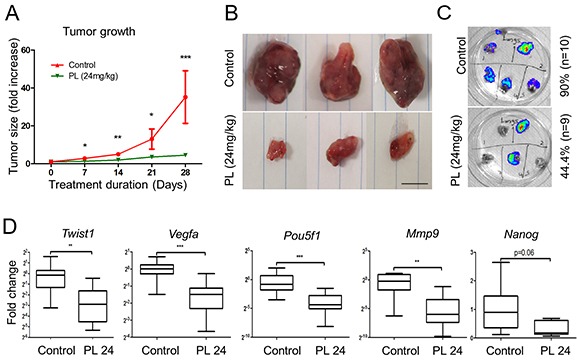
Piperlongumine inhibits tumor growth and metastases formation and decreases expression levels of EMT and angiogenesis markers *in vivo* **A.** Nude female mice bearing MTT-Luc tumors were treated with 24 mg/kg/day PL (n=9) for 28 days. The control group was treated with vehicle (n=10). Tumor growth was assessed once a week. The graph shows a significant inhibition of tumor growth at all time points of treatment in the treated group (green) compared to control (red) animals. The graph represents data from 9 (treated) and 10 (vehicle) mice assessed at specific time points as mean +/− SEM. Statistical analysis was performed by Mann Whitney, U-test, at each time point. **B.** Representative tumors from control mice compared to the tumors from animals treated with PL. Scale bar: 1cm. **C.** Lungs were resected and subjected to bioluminescence imaging. The presence of lung metastases in treated mice was 46% lower than in control group. **D.** mRNA expression levels of *Pou5f1, Twist1, Vegfa, Mmp9,* and *Nanog* in tumors from both treated and control groups were assessed by quantitative real-time PCR. The target gene transcript levels were normalized to *Actb*. The box and whiskers graphs represent data from control (n=10) and treated (n=9) groups. *P<0.05; ** P<0.01; ***P<0.001, Mann Whitney, U-test. PL: piperlongumine; *Mmp9*: matrix metallopeptidase 9; *Pou5f1*: POU domain, class 5, transcription factor 1.

To assess the effect of PL on tumors *in vivo*, we used a previously described allograft model of PHEO [35]. The mice were treated with PL (24mg/kg/day) or vehicle for 28 days. The mice did not demonstrate any adverse drug-related physiological or behavioral side effects (Supplementary Figure S3A). PL significantly inhibited tumor growth from the first week of treatment, and the growth remained delayed until the end of the study (Figure 4A, 4B). After sacrificing the animals, we excised the lungs and livers, the two most common metastatic sites of PHEO/PGL cells [35]. In addition, we performed a pathological evaluation of the animals in order to find potential metastases or local invasion in the direct environment of primary tumors. Liver metastases were found at similar frequency in both groups. In the treated group, the number of lung metastases was significantly reduced compared to control (44% vs. 90%) (Figure 4C). Additional metastases in the peritoneum or near the primary tumors were found in 80% of non-treated mice but in only 22% of the treated animals (Supplementary Table S1).

**Figure 5:**
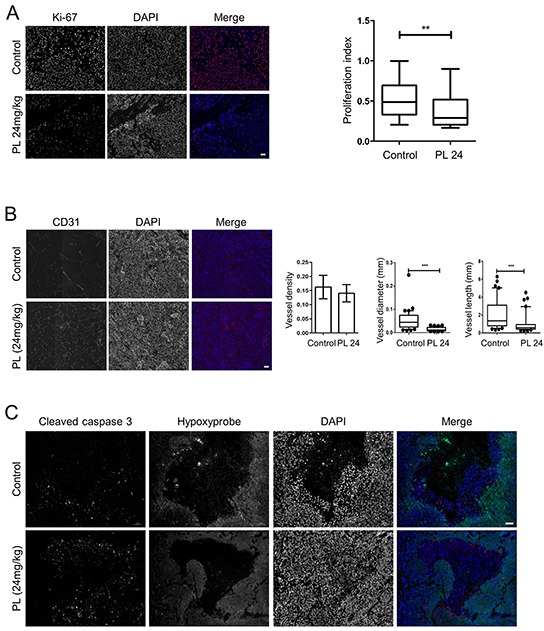
Piperlongumine decreases tumor cell proliferation and angiogenesis and increases apoptotic cell death *in vivo* **A.** Ki-67 staining demonstrates a significantly lower proliferation rate of cells in tumors from treated mice than in control animals. The representative images (treated: n=5, control: n=5) show individual staining of Ki-67 and DAPI and merged images of the two; Ki-67 (red), DAPI (blue). Magnification: 20x. Scale bar: 50μM. The box and whiskers graph represents data from control and treated groups. **B.** CD31 staining shows significantly reduced tumor vessel length and diameter in treated animals (n=5) than in control mice (n=5). The representative images show individual staining of CD31 and DAPI and merged images of the two; CD31 (red), DAPI (blue). Magnification: 20x. Scale bar: 50μM. Error bars for the density: +/−SEM. For length and width, the box and whiskers graphs represent data from control and treated animals. **C.** The tumors from both groups (treated: n=5, control: n=5) were stained for cleaved caspase 3 and hypoxic regions. The representative images show separate staining of cleaved caspase 3, hypoxic regions, and DAPI. In the merged image, green represents hypoxic regions, red is cleaved caspase 3, and blue is DAPI. Magnification: 20x. Scale bar: 50μM. ** P<0.01, ***P<0.001, Mann Whitney, U-test. PL: piperlongumine.

We also evaluated the expression of EMT and angiogenesis markers in the two groups. Transcript level analysis showed a significant decrease in the expression of *Twist1*, *Pou5f1, Mmp9* and *Vegfa* in treated group; transcription levels of *Nanog* were almost significantly decreased (Figure 4D). We also performed staining for Ki-67 to assess the cell proliferation, and found a significant decrease in the proliferation index in the treated group (Figure 5A), suggesting that PL inhibits tumor cell proliferation. In order to analyze the vasculature of the tumors we performed CD31 staining and while there was no significant difference in vessel density between the two groups, vessel diameter and length were significantly reduced in treated animals (Figure 5B).

To confirm that the effect of PL on tumor growth and size was promoted by apoptosis, we co-stained the tumors for cleaved caspase 3 and hypoxic regions. We demonstrated significantly higher levels of cleaved caspase 3 in treated mice (Figure 5C; Supplementary Figure S3B). The staining signal was predominantly localized to the regions with incorporated hydroxyprobe, suggesting that hypoxia contributes to an increased rate of cell death, which was also indicated by the fact that hypoxic areas were significantly larger in the control than in treated animals (Supplementary Figure S3C). To further confirm that ROS-associated DNA damage was inducing cell death in the tumors, we analyzed protein levels of cleaved RIP1 and γH2A.X and observed higher levels of both proteins in the treated group of animals (Supplementary Figure S3D). Elevated levels of necrosome in treated animals also suggest a necroptosis-inducing effect of PL *in vivo* (Supplementary Figure S3E). In addition, we measured ROS levels in tumor cells from animals treated with PL for one week and a non-treated group. We observed an increase of ROS in the treated group (Supplementary Figure S3F), suggesting that PL exerts its cytotoxic effect via ROS induction also *in vivo*.

We did not observe any change in autophagy-associated proteins in the animal tumors (Supplementary Figure S3G). On the other hand, levels of phospho-ERK were higher in treated animals, suggesting involvement of the ERK pathway in the execution of cell death (Supplementary Figure S3G). The data suggest that PL exhibits both pro-apoptotic and –necroptotic effects *in vivo*, which leads to tumor growth inhibition and reduction in the number of metastases.

## DISCUSSION

In the present work, we studied the cytotoxic effect of PL on PHEO cells and found that PL promotes PHEO cell death through ROS-induced apoptosis and necroptosis. Moreover, the cytotoxic effect of PL was significantly increased in cells exposed to hypoxia as well as in the PHEO/PGL cell model lacking SDHB protein, resembling the pseudohypoxic phenotype of *SDHB*-related tumors [36]. In addition, PL significantly reduced pro-angiogenic and metastatic properties of the cells *in vitro* and *in vivo*. The findings make PL an attractive therapeutic option that works through mechanisms different from those already reported in PHEOs/PGLs.

Despite an extensive search for effective drugs for metastatic PHEOs/PGLs, there is currently no satisfactory treatment of this disease. Although several molecules have been suggested as possible treatment targets, including topoisomerases, mTOR and HSP90, today, CVD chemotherapy is the most promising therapy for patients with *SDHB* mutations, but ultimately its use is limited due to the development of resistance or severe cytotoxic effects of CVD [37–39]. Thus, it is of great importance to identify new ways to treat metastatic PHEOs/PGLs and to overcome resistance mechanisms of PHEO/PGL cells.

For the first time, we evaluated the effects of PL to induce ROS overproduction in mouse-derived PHEO cells. To our best knowledge, this approach has never been investigated in PHEOs/PGLs. One important feature of cancer in general is a high level of intracellular ROS. This characteristic is even more prominent in the *SDHB*-related PHEOs/PGLs [40]. This led us to the hypothesis that further increasing intracellular ROS levels would disrupt the tightly regulated equilibrium that was protecting the cells from the cytotoxic effects of ROS, and thus promote cancer cell death.

In the present study we show that in PHEOs/PGLs, like in other cancer models, treatment with PL leads to ROS-induced apoptosis [26, 41, 42]. While PL has been shown to activate autophagy in several cancer cell types [21, 43, 44], this pathway is not affected in MPC/MTT cells. On the other hand, we demonstrate for the first time that PL induces necroptosis, a form of controlled necrosis associated with mitochondrial fission and the release of ROS [45], which might further magnify the effect of PL. Necrosis has also been reported as a consequence of PL treatment in selected leukemia cell lines [46]. Our results are a proof of concept that targeting ROS production in PHEOs/PGLs could be effective in the treatment of these tumors.

A further challenge in the treatment of PHEOs/PGLs is the presence of hypoxia. Cells exposed to hypoxic conditions exhibit different behavior from those under normal oxygen levels. Hypoxia activates numerous pathways, which are silent in normoxic conditions, resulting in resistance to chemo- and radiotherapy [1]. Our study shows that treatment of PHEO cells with PL in hypoxia leads to higher levels of ROS and apoptotic and necroptotic markers when compared to normoxic conditions, resulting in a magnified cytotoxic effect of PL on these cells. We observed similar results using neuroblastoma cell line, where the effect of PL was more prominent in pseudohypoxic cells without the SDHB protein. We also demonstrated activation of the MAPK pathway, mainly phospho-ERK1/2, which was increased 8-fold in cells treated with PL in hypoxia (Supplementary Figure S2B). PL inhibits tumor angiogenesis and EMT processes that are promoted by low oxygen levels [47]. To our best knowledge, the only proteasome-inhibitor that has been shown to be more effective in hypoxia is bortezomib [48]. However, in another study the efficacy of bortezomib did not change in hypoxic conditions [49], suggesting the effect is cell type-dependent. Kretowski *et al.* did not describe the mechanisms leading to a higher number of apoptotic cells in hypoxia, though they do describe an increase in HIF-1α levels in cells treated with bortezomib [48]. These findings are in accordance with ability of bortezomib to inhibit proteasomal activity, preventing HIF-1α from being degraded. However, PL most likely exerts its effect through a different mechanism, as we did not detect any change in the ubiquitin profile after treatment with PL in MPC cells.

Conflicting results have been observed concerning ROS and hypoxia, as low oxygen levels have been shown to both lower as well as elevate intracellular ROS levels [50], with the latter being the case in MPC cells. Similarly, ROS have been reported to stabilize the HIF-α subunit, while in others studies its expression was decreased [50–52]. Hypoxia suppresses and also activates the apoptotic pathway, with our results confirming a pro-apoptotic effect of hypoxia [12]. The hypoxia-associated apoptosis can be regulated by both HIF-1 and HIF-2 [12, 53]. In our models, the induction of cell death in hypoxia is independent of HIF-2, as the models used are defective for HIF-2α. One of the mechanisms leading to the enhanced cytotoxic effect of PL in hypoxia is most likely its ability to further increase the elevated ROS levels, reaching the cytotoxic threshold of PHEO cells at lower concentrations of the drug. ERK1/2 may also play an important part in this process, since its role in cell death induction is well established [33]. However, the exact mechanisms of PL-induced cell death associated with hypoxia will require further investigation.

Preliminary studies evaluating the role of EMT in metastatic PHEOs/PGLs suggest that targeting EMT might be beneficial in the treatment of the disease, especially *SDHB*-related tumors [36, 54]. Our results strongly support this hypothesis, as PL significantly reduced the expression of several EMT markers as well as extracellular matrix-modulating effectors described in angiogenesis. *In vivo* this reduction led to a significant decrease in tumor growth and the number of metastases.

In the current study we used PHEO cells derived from heterozygous *Nf1* knock-out mice. The cell line resembles the human disease associated with *NF1* mutations, and is characterized by production of catecholamines, tyrosine hydroxylase, and phenylethanolamine N-methyltransferase [55, 56]. Based on the common features of tumors in cluster 2, which includes tumors with mutations in *NF1*, we believe that the effect mediated by PL in MPC cells could result in a similar outcome in other tumors in this cluster, regardless of the type of mutation. Moreover, based on our results showing that the cytotoxic effect of PL is stronger in hypoxia and in cells lacking SDHB, we propose that PL could also potentially be effective in cluster 1 tumors due to their pseudohypoxic character.

In summary, in the present study we show that PL mediates its cytotoxic effect on PHEO cells by increasing ROS levels, resulting in apoptosis, which is further enhanced in hypoxic conditions. In addition, this is the first report of activation of necroptosis by PL, a type of cell death also increased under hypoxic conditions. Moreover, for the first time, we observed necroptosis in PHEOs/PGLs, which is an important finding as resistance to apoptosis is one of the hallmarks of cancer [57] and therefore, other options need to be investigated in order to overcome this resistance. We also demonstrate that PL is effective in the inhibition of angiogenesis and metastases formation. Overall, our results demonstrate that PL is a promising agent for the treatment of PHEOs/PGLs, and because of its enhanced efficacy in hypoxia, it has a high potential to also be effective in *SDHB*-related tumors.

## MATERIALS AND METHODS

### Cell lines

In this study, we used the mouse pheochromocytoma cell lines MPC/4/30PRR, MTT, MTT-Luc and mouse neuroblastoma cells N2a (CCL-131; ATCC, Manassas, VA, USA). MPC/4/30PRR (abbreviated as MPC) are cells derived from a pheochromocytoma from heterozygous *Nf1* knockout mouse [56], MTT (mouse tumor tissue) are cells derived from a liver metastasis formed after the injection of MPC cells. The cell line exhibits a more aggressive phenotype than MPC cell line [55]. This cell line was also used to create MTT-Luc cells to study the disease in animal models using *in vivo* imaging [35]. The cells were maintained in DMEM supplemented with 10% fetal bovine serum, 5% horse serum, and antibiotic/antimycotic (Life Technologies Carlsbad, CA, USA). For experiments in hypoxia, the cells were incubated at 1% O_2_ and 5% CO_2_ using the InvivO_2_ Hypoxia 300 workstation (Ruskinn Technology Ltd, Bridgend, UK).

### Reagents

Piperlongumine (PL) was purchased from Selleckchem (Houston, TX, USA); stock solutions were stored at −20°C and thawed prior to use. The same protocol was used to prepare Z-DEVD-FMK (R&D Systems, Inc, Minneapolis, MN, USA), Z-VAD-FMK, (Selleckchem, Houston, TX, USA), 3-methyladenine, superoxide dismutase, superoxide dismutase-PEG, 2′,7′-dichlorofluorescin diacetate (DCF-DA), siRNA targeting mouse *Sdhb* and *EGFP* (Sigma-Aldrich, St. Louis, MO, USA), and necrostatin-1 (Millipore, Billerica, MA, USA). Ascorbic acid was purchased from Sigma-Aldrich (St. Louis, MO, USA) and stock solution was prepared prior to use.

### siRNA transfection

1.5 × 10^6^ N2a cells were transfected with 20nM siRNA against *Sdhb* using the Lipofectamine RNAiMAX, diluted in Opti-MEM medium (Life Technologies Carlsbad, CA, USA) and plated in a 10cm dish. Control cells were transfected with siRNA targeting *EGFP* at the same concentration. The following day, the cells were trypsinized and plated for further experiments.

### Western blotting and RIP3 immunoprecipitation

The western blotting was performed as reported previously [37]. RIP3 immunoprecipitation was performed using the Pierce Co-Immunoprecipitation kit according to the manufacturer's instructions (Thermo Scientific, Inc., Pittsburgh, PA, USA). The primary antibodies included anti-cleaved caspase 3 (5A1E; 9664), anti-caspase 3 (8G10; 9665), anti-cleaved PARP (9544), anti-LC3A/B (4108), anti-LAMP1 (C54H11; 3243), anti-γH2A.X (2577), anti-RIP1 (D94C12; 3493), anti-histone 3 (D1H2; 4499; Cell Signaling Technology, Danvers, MA, USA), anti-p62 (ab56416, Abcam, Cambridge, UK), anti-ubiquitin (FK2; ST1200; Millipore, Billerica, MA, USA), anti-RIP3, anti-β-tubulin (D66; T0198), anti-RIP1 (334640; MAB3585, R&D Systems, Minneapolis, MN, USA), anti-RIP3 (R4277) and anti-SDHB (HPA002868; Sigma-Aldrich, St. Louis, MO, USA). For the detection of phospo-kinases, the Proteome Profiler Human Phospho-Kinase Array Kit (R&D Systems, Inc, Minneapolis, MN, USA) was used and the samples were analyzed according to the manufacturer's instructions.

### Cell viability assay

Cell viability was assessed by (3-(4, 5-dimethylthiazolyl-2)-2, 5-diphenyltetrazolium bromide) (MTT) assay (Sigma-Aldrich, St. Louis, MO, USA), according to the manufacturer's instructions and as reported previously [37]. In addition, cytolysis was investigated by measuring lactate dehydrogenase (LDH) release using an LDH kit (Roche Diagnostics) according to the manufacturer's recommendations [58].

### Cell migration and invasion assay

The migration assay was performed as reported previously [59]. The number of migrated cells was assessed using fluorescence microscope and analyzed using ImageJ software (NIH, Bethesda, MD, USA). The Invasion assay was performed the same way, with transwell chambers coated with matrigel (Trevigen, Gaithersburg, MD, USA).

### Intracellular ROS measurement

MPC cells were plated and incubated overnight prior to treatment with the indicated PL concentrations for 3h. For hypoxic conditions, cells were incubated at 1% O_2_ for 12h prior to the treatment. After the treatment, DCF-DA (Sigma-Aldrich, St. Louis, MO, USA) was added to the cells at a working concentration of 10μM for 30 minutes, and the samples were analyzed by fluorescence microscopy (Zeiss, Oberkochen, Germany). Alternatively, the cells were trypsinized and incubated with 10μM DCF-DA at 37°C for 30 minutes and analyzed by flow cytometry (BD, San Diego, CA, USA) according to the manufacturer's instructions. Similarly, the cells were treated with MitoSOX Red Mitochondrial Superoxide Indicator (ThermoFisher Scientific, Waltham, MA USA) according to the manufacturer's instructions and analyzed by flow cytometry.

### RNA extraction and real time quantitative PCR

mRNA was extracted using the RNAQuick-micro kit (Zymo Research, Irvine, CA, USA). mRNA was quantified by Nanodrop (Thermo Scientific, Inc., Pittsburgh, PA, USA) and 1μg was reverse-transcribed by the SuperScript III RT kit (Life Technologies Carlsbad, CA, USA) according to the manufacturer's instructions. qPCR was performed in ABI Viia-7 (Life Technologies Carlsbad, CA, USA) using TaqMan probes (Life Technologies Carlsbad, CA, USA). The probes included Mm00441533_g1, Mm03053917_g1, Mm01247357_m1, Mm01281449_m1, Mm04208233_g1, Mm00441531_m1, Mm02384862_g1. The amount of mRNA detected was normalized to control *Actb* mRNA values. Relative gene expression is a percentage of the ratio value obtained in non-treated cells or animals from the same experiment.

### Apoptosis assay

MPC cells treated with or without PL for 24h in normoxia or hypoxia were harvested and stained using the FITC-Annexin V Apoptosis Detection Kit I (BD, San Diego, CA, USA) and analyzed by flow cytometry according to the manufacturer's instructions.

### Animal experiments and bioluminescence imaging

1.5 × 10^6^ MTT-Luc cells were injected subcutaneously in the right lower dorsal side of female athymic nude mice (Charles River, Germantown, MD, USA). The experimental group consisted of 6-week-old mice (n=19) housed in a pathogen-free facility. After 10 days to allow for tumor cells to engraft, we started a daily injection with 24mg/kg PL (n=9) or vehicle (0.06% DMSO in PBS) (n=10). The animals were imaged weekly by bioluminescence as reported previously [59]. The tumor volume was calculated using the formula V=width^2 × length, where the width was the shorter dimension and length the longer dimension of the tumor. Before the last imaging, mice were injected with 60mg/kg of Hydroxyprobe (pimonidazole HCl) (Hydroxyprobe, Inc, Burlington, MA, USA) and luciferin. All animal studies were conducted in accordance with the principles and procedures outlined in the NIH Guide for the Care and Use of Animals and approved by the NIH Animal Care and Use Committee.

### Immunofluorescence staining

The tumors from the animals were embedded in paraffin and slides with 5μm thick tissue recuts were prepared. The tissue was deparaffinized and rehydrated prior to antigen retrieval in citrate sodium buffer (pH 6.0) for 20 minutes. The sections were incubated with primary antibody for one hour at room temperature, followed by one-hour incubation with a secondary antibody labeled with DyLight 594 or 488 (Vector Laboratories, Burlingame, CA, USA). The nuclei were labeled with NucBlue Live ReadyProbes Reagent (Life Technologies Carlsbad, CA, USA) for 10 minutes and mounted with Mowiol mounting solution (Millipore, Billerica, MA, USA). The staining was analyzed by fluorescence microscope (Zeiss, Oberkochen, Germany). The primary antibodies used included anti-Ki67 (ab15580) and anti-CD31 (ab28364) (Abcam, Cambridge, UK). When the tissue was co-stained to indicate hypoxic regions, a 30-minute incubation step with FITC-MAb1 (Hydroxyprobe, Inc, Burlington, MA, USA) at room temperature was added after the staining with secondary antibody. FITC-MAb1 binds to adducts formed by hypoxia-activated pimonidazole with thiol groups in proteins, peptides, and amino acids.

### Statistics

Statistical analyses were performed using GraphPad Prism software, non-parametric Mann-Whitney U test (GraphPad Software, San Diego, CA, USA).

## SUPPLEMENTARY FIGURES AND TABLES



## Abbreviations

*Actb*: beta actin
CVD: cyclophosphamide+vincristine+dacarbazine
γH2A.X: gamma H2A histone family, member X
EMT: epithelial-mesenchymal transition
ERK: extracellular signal-regulated kinase
GSK: glycogen synthase kinase
HIF: hypoxia-inducible factor
JNK: c-Jun N-terminal protein kinase
LDH: lactate dehydrogenase
3MA: 3-methyladenine
MAPK: mitogen-activated protein kinase
*Mmp9*: matrix metallopeptidase 9
MPC cells: mouse pheochromocytoma cells
mTOR: mammalian target of rapamycin
MTT cells: mouse tumor tissue cells
Nec1: necrostatin 1
*NF1*: neurofibromin 1
PARP: poly (ADP-ribose) polymerase
PHEOs/PGLs: pheochromocytomas/paragangliomas
PI3-K: phosphatidylinositol 3-kinase
PL: piperlongumine
*Pou5f1*: POU domain, class 5, transcription factor 1
RIP: receptor-interacting protein kinase
ROS: reactive oxygen species
*SDHB*: subunit B of succinate dehydrogenase
SOD: superoxide dismutase
SOD-PEG: superoxide dismutase-polyethylene glycol
