# Supplementary material for: Hypoxia potentiates the cytotoxic effect of piperlongumine in pheochromocytoma models
Source: Oncotarget. 2016 May 26;7(26):40531–45. doi: 10.18632/oncotarget.9643 (PMC5130026; doi:10.18632/oncotarget.9643)
Supplement: Supplementary file 1 [file oncotarget-07-40531-s001.pdf]

SUPPLEMENTARY FIGURES

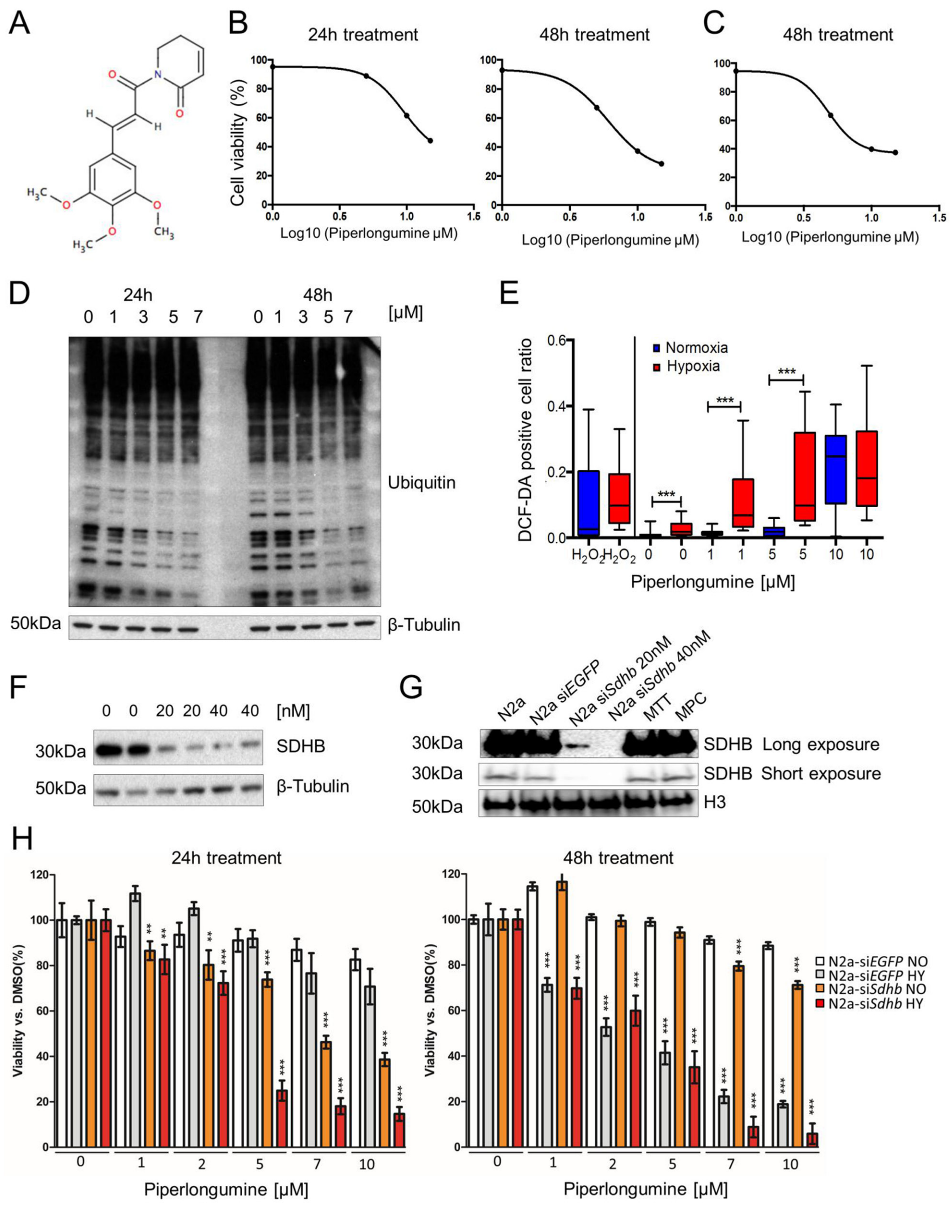

Supplementary Figure S1: Piperlongumine exhibits its cytotoxic effect on PHEO cell models by increasing ROS levels. **A.** The chemical structure of PL. **B.** MPC cells were treated with 0, 1, 5, 10 and 15μM PL for 24 and 48 hours. Cell viability was assessed by MTT assay. **C.** MTT cells were treated with 0, 1, 5, 10 and 15μM PL for 48 hours. Cell viability was assessed by MTT assay. **D.** MPC cells were treated with indicated concentrations of PL for 24 and 48 hours. Total protein lysates were subjected to Western blot and ubiquitin profile was analyzed. β-tubulin was used as a loading control. The representative image (n=3) is shown. **E.** MPC cells were treated with 1, 5 and 10μM PL at 21% and 1% O_2 for 3 hours. ROS level increase in treated cells was evaluated by fluorescence microscope. The box and whiskers graphs represent data from three independent experiments. **F.** N2a cells were transfected with indicated concentrations of siRNA targeting *Sdhb*; siRNA targeting *EGFP* was used as a control. Total cell lysates were analyzed by Western blot for SDHB. β-tubulin was used as a loading control. **G.** Total cell lysates from N2a cells with silenced *Sdhb* and control cells were analyzed by Western blot for SDHB. To compare SDHB protein levels, MPC/MTT cells were used. Histone 3 was used as a loading control. **H.** si*Sdhb*-N2a and si*GFP*-N2a cells were treated with 0, 1, 2, 5, 7, and 10μM PL at 21% and 1% O_2 for 24 and 48 hours. Cell viability was assessed by MTT assay. **P<0.01, ***P<0.001, Mann Whitney, U-test.

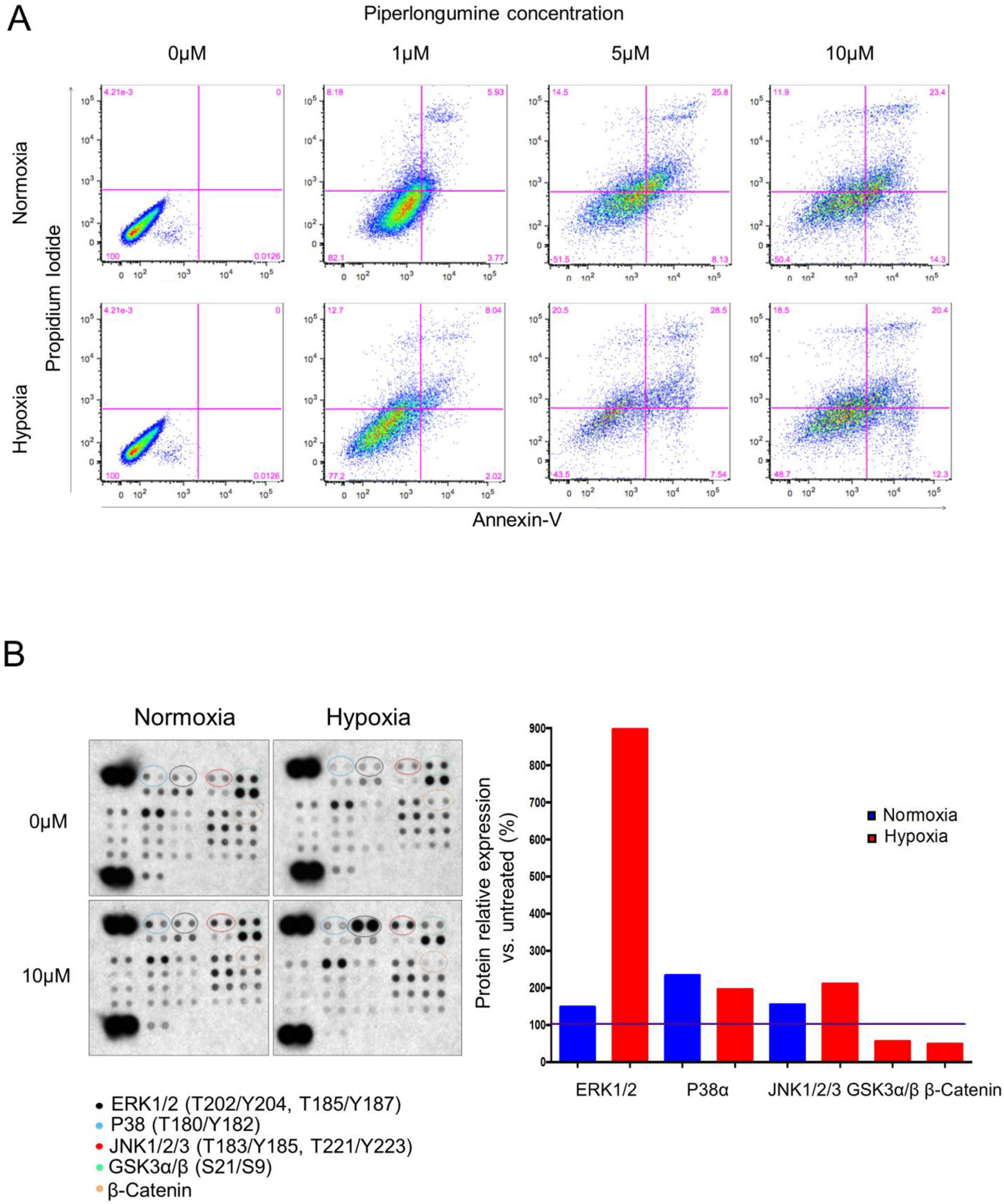

Supplementary Figure S2: The cytotoxic effect of piperlongumine is magnified in hypoxia. A. MPC cells were treated with 0, 1, 5 and 10μM PL at 21% and 1% O_2 for 24 hours. The cells were stained for Annexin V and Propidium Iodide and analyzed by Flow Cytometry. **B.** MPC cells were treated with 10μM PL at 21% and 1% O_2 for 24 hours. Total cell lysates were analyzed by proteome profiler array kit for phosphorylated kinases. The graph represents the total signal of the duplicates from the image on the left.

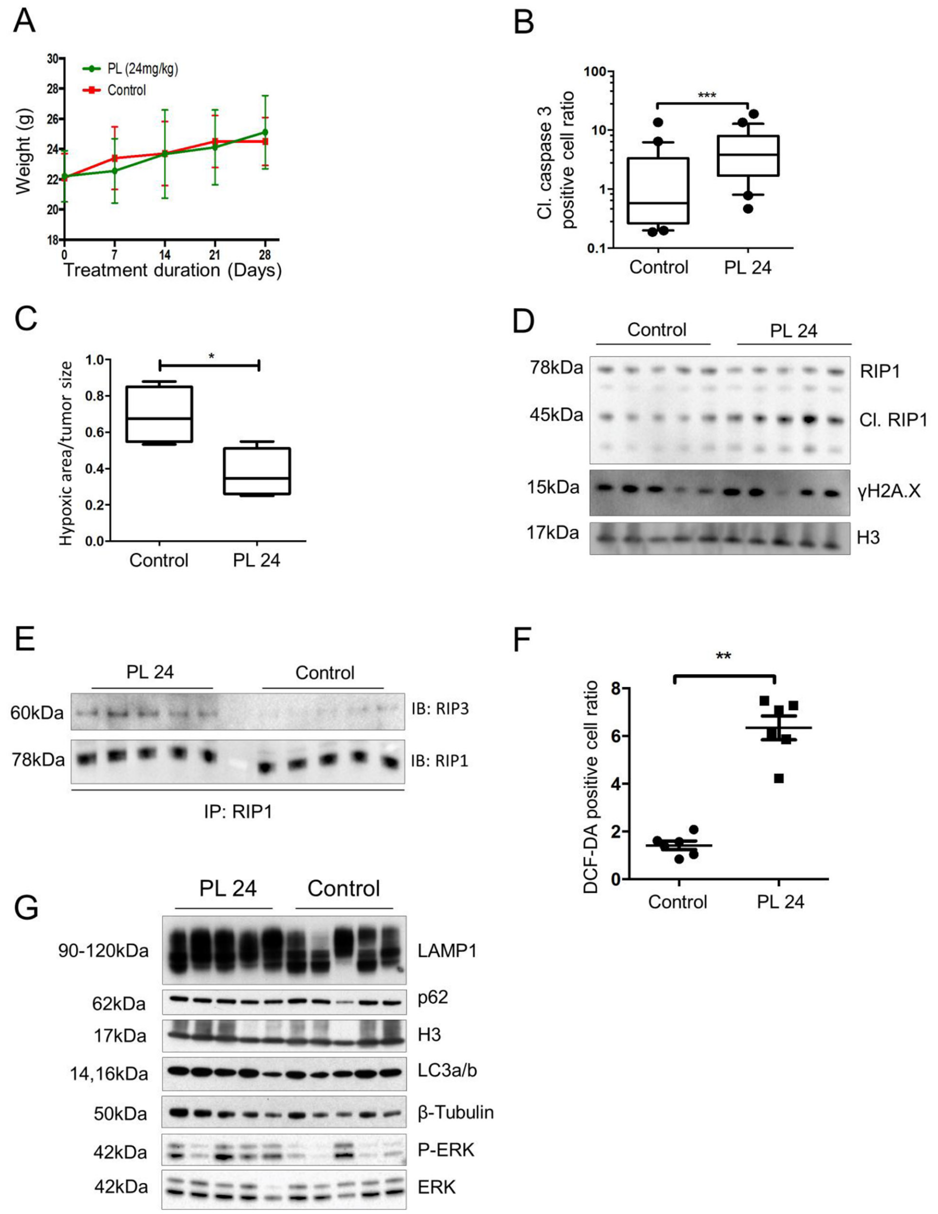

Supplementary Figure S3: Piperlongumine exhibits its cytotoxic effect on pheochromocytoma *in vivo*. A. The graph represents mice weight, in both treated (green) and control (red) groups, weighed and recorded on a weekly basis. **B.** The tumors from both groups were stained for cleaved caspase 3. The box and whiskers graph represents data from control and treated groups. **C.** The tumors from both groups were stained for hypoxic regions. The graph represents ratio of hypoxic area to the size of a tumor. The box and whiskers graph represents data from control and treated groups. **D.** Total proteins were extracted from tumors from treated (n=5) and control (n=5) groups and were subjected to Western blot with antibodies against cleaved RIP1 and γH2A.X. Histone 3 was used as a loading control. **E.** RIP1-IP was performed on cell lysates from tumors from treated and control groups and probed from RIP3 (top lane) and RIP1 (bottom lane). **F.** Mice were treated with 24mg/kg PL or vehicle per day for one week. ROS production levels were analyzed by Flow Cytometry. The box and whiskers graph shows MFI for DCF-DA positive cells from 6 tumors in each group. **G.** Total proteins were extracted from tumors from treated (n=5) and control (n=5) groups and were subjected to Western blot with antibodies against p62, LAMP1, LC3a/b phospho-ERK and ERK. β-tubulin and histone 3 were used as loading controls. $^*P<0.05$, $^{**}P<0.01$; Mann Whitney U-test.

Supplementary Table S1: Piperlongumine inhibits metastatic potential of pheochromocytoma *in vivo*

	Lungs	Liver	Other
Treated group	44.4%	11.1%	22.2%
Control group	90%	10%	80%

The table summarizes the findings of pathology analysis of the allograft bearing mice treated with 24 mg/kg PL per day and control animals. The percentage values represent metastases found in the liver, lungs, peritoneum or in the proximity to the primary tumors.
